# Stem Cells for Cartilage Repair: Preclinical Studies and Insights in Translational Animal Models and Outcome Measures

**DOI:** 10.1155/2018/9079538

**Published:** 2018-02-05

**Authors:** Melissa Lo Monaco, Greet Merckx, Jessica Ratajczak, Pascal Gervois, Petra Hilkens, Peter Clegg, Annelies Bronckaers, Jean-Michel Vandeweerd, Ivo Lambrichts

**Affiliations:** ^1^Department of Morphology, Biomedical Research Institute, Faculty of Medicine and Life Sciences, Hasselt University, Campus Diepenbeek, 3590 Diepenbeek, Belgium; ^2^Department of Veterinary Medicine, Integrated Veterinary Research Unit-Namur Research Institute for Life Science (IVRU-NARILIS), Faculty of Sciences, University of Namur, 5000 Namur, Belgium; ^3^Department of Musculoskeletal Biology, Faculty of Health and Life Sciences, University of Liverpool, Leahurst Campus, Neston CH64 7TE, UK

## Abstract

Due to the restricted intrinsic capacity of resident chondrocytes to regenerate the lost cartilage postinjury, stem cell-based therapies have been proposed as a novel therapeutic approach for cartilage repair. Moreover, stem cell-based therapies using mesenchymal stem cells (MSCs) or induced pluripotent stem cells (iPSCs) have been used successfully in preclinical and clinical settings. Despite these promising reports, the exact mechanisms underlying stem cell-mediated cartilage repair remain uncertain. Stem cells can contribute to cartilage repair via chondrogenic differentiation, via immunomodulation, or by the production of paracrine factors and extracellular vesicles. But before novel cell-based therapies for cartilage repair can be introduced into the clinic, rigorous testing in preclinical animal models is required. Preclinical models used in regenerative cartilage studies include murine, lapine, caprine, ovine, porcine, canine, and equine models, each associated with its specific advantages and limitations. This review presents a summary of recent *in vitro* data and from *in vivo* preclinical studies justifying the use of MSCs and iPSCs in cartilage tissue engineering. Moreover, the advantages and disadvantages of utilizing small and large animals will be discussed, while also describing suitable outcome measures for evaluating cartilage repair.

## 1. Introduction

Articular cartilage covers the ends of the bone; due to its slightly compressible and elastic nature and lubricated surface, it provides the joint with shock absorption and lubrication [1, 2]. Hyaline cartilage is comprised of 95% extracellular matrix (ECM) (dry weight) and only 5% of sparsely distributed chondrocytes [3]. This matrix primarily consists of type II collagen and proteoglycans (PGs). Negatively charged glycoproteins are able to attract water, allowing the cartilage to resist compressive forces [4]. Despite the fact that chondrocytes only make up about 5% of hyaline cartilage tissue, they are integral for cartilage function and homeostasis [4]. These cells are of mesenchymal origin and are responsible for synthesizing cartilage ECM [3]. Hyaline cartilage is an avascular tissue which, in part, explains the limited regeneration capacity following injury. The lack of vasculature makes it difficult for progenitor cells to be recruited to the site of injury and hinders the supply of nutrients necessary for tissue regeneration [1, 5].

Cartilage loss can occur as a consequence of traumatic injury, leading to focal defects or through chronic degeneration. Both partial thickness and full thickness cartilage defects occur [6]. Since full thickness lesions extend into the subchondral bone, they have access to bone marrow cells and therefore have a higher probability of spontaneous regeneration than partial thickness lesions, which only involve the avascular cartilage tissue [6]. Eventually, cartilage defects will lead to activity-related pain, swelling, and decreased mobility and will frequently progress to osteoarthritis [1, 7]. In the United States alone, over 27 million adults suffer from osteoarthritis, leading to a substantive clinical and financial burden [8, 9].

There are currently no drugs available to effectively heal cartilage defects. When cartilage defects develop into osteoarthritis, the condition can only be managed by a multidisciplinary approach including pharmacotherapy, physiotherapy, or joint replacement surgery [10]. However, several surgical interventions can be performed in order to prevent progression towards osteoarthritis [1]. Current techniques include arthroscopic lavage and debridement, microfracture induction, and autologous chondrocyte implantation [11]. Although these techniques have been proposed to restore normal joint function and minimize further degeneration, they often do not offer a long-term clinical solution. There is a clinical need to develop regenerative medicine approaches to permanently restore articular cartilage [11].

Both adult mesenchymal stem cells (MSCs) and induced pluripotent stem cells (iPSCs) are promising stem cell sources to achieve cartilage regeneration [5, 7, 12–14]. However, the use of adult MSCs still faces considerable challenges such as cell senescence and donor variability [7, 15]. iPSCs may provide a suitable alternative in order to overcome the limitations of adult MSCs [7]. iPSCs possess unlimited self-renewal and pluripotency, similar to embryonic stem cells (ESCs), but lack the ethical concerns associated with the use of ESCs [1]. However, it remains to be determined whether differentiated iPSCs are able to form a bona fide cartilage [1]. Furthermore, more research is required to alleviate any concerns for tumorigenic effects before this technology can progress to preclinical and clinical usage [16, 17]. Before any of these possible treatment options can be introduced into the clinic, they first have to be tested in suitable and translational animal models [9]. A wide variety of animal models is available to investigate cartilage regeneration ranging from small animal models, such as mice and rats, to larger animals such as canine, porcine, caprine, ovine, and equine models. Smaller animal models are cost-effective and easy to house and offer a variety of genetically modified or immunocompromised strains. However, due to their small joint size and thin cartilage, their translational value is limited [9]. Larger animal models on the other hand more accurately approximate the human situation but are associated with greater logistical, financial, and ethical considerations [9].

In this review, recent *in vitro* data and preclinical studies justifying the use of MSCs and iPSCs in cartilage tissue engineering are summarized. Since preclinical studies require translational animal models, the advantages and disadvantages of small and large animal models will be discussed, while also focusing on suitable outcome measures for evaluating cartilage repair.

## 2. *In Vitro* Evidence of Chondrogenic Differentiation of Stem Cells

For stem cell-based cartilage regeneration, MSCs are of particular interest because, in comparison to chondrocytes, they have high availability and both easy isolation and expansion [18]. In addition, their *in vitro* chondrogenic differentiation has been proven [19]. More recently, *in vitro* studies on iPSCs indicated promising results for their use in cartilage repair [20, 21]. However, a number of challenges have to be overcome and further optimization is still needed before both stem cell types can be used as a safe and effective therapeutic option for promoting cartilage repair [1, 14, 22–24].

### 2.1. Mesenchymal Stem Cells

Adult MSCs were first identified in bone marrow [25], but afterwards, other MSC niches have been discovered in both adult and fetal tissues, including adipose tissue [26], placenta [27], umbilical cord [28], dental pulp [29, 30], and peripheral blood [31], and in the synovial membrane [32]. As defined by the International Society for Cellular Therapy (ISCT), MSCs must be able to differentiate into chondrocytes under specific *in vitro* conditions [33]. In addition, MSCs possess additional properties making them a suitable cell source for cartilage regeneration. High cell numbers can be produced, and the immunomodulatory characteristics of MSCs allow for their allogeneic use [34].

Pellet and monolayer cultures are the two main culture systems that have been developed to study *in vitro* chondrogenic differentiation. The 3D pellet system is the most representative *in vitro* model for the condensation of mesenchymal cells that is observed during the initiation phase of chondrogenesis in the process of endochondral ossification [35, 36]. Moreover, cocultures with chondrocytes in both 2D and 3D culture systems could push MSCs towards the chondrogenic lineage [37–39] and growth factors, such as insulin-like growth factor (IGF) [40] and members of the fibroblast growth factor (FGF) [41] and transforming growth factor-beta (TGF-*β*) [42–44] families, can be added to the differentiation medium to enhance chondrogenic differentiation. Additionally, the chondrogenic differentiation potential of MSCs and the production of ECM proteins can also be stimulated by combining MSCs and biomaterials in 3D scaffolds [45–52] or by manipulating the oxygen tension [53].

*In vitro* studies mainly focus on bone marrow-derived MSCs (BM-MSCs), followed by MSCs derived from adipose tissue and synovial membrane because of their easy isolation and close proximity to cartilage and joints, respectively [16]. A correlation between the chondrogenic potential of MSCs and their tissue source has been suggested. BM-MSCs showed a superior chondrogenic differentiation capacity compared to MSCs from other origins [54–56]. These differences might be explained by variations in gene expression and pathway activation [57]. Therefore, an adapted differentiation protocol could compensate for lower chondrogenic differentiation capacities [57, 58].

Despite their promising chondrogenic potential *in vitro*, several challenges are linked to the use of MSCs in cartilage regeneration. The most common issue is terminal differentiation towards hypertrophic cells [36]. Moreover, mineralization and vascularization have also been reported after transplantation [35, 59]. In addition, cartilage tissue derived from *in vitro* differentiated MSCs resembles fibrocartilage with inferior mechanical properties and healing capacity [22]. Another limitation is the inter- and intradonor heterogeneity of MSCs which could influence chondrogenic differentiation potential of cells [60], depending on comorbidities, tissue source, and culture methods [24]. Furthermore, serial passaging, needed to obtain sufficient cell numbers for *in vivo* studies, has been reported to affect chondrogenic differentiation of BM-MSCs [61]. Finally, supplementation of the culture media with high and repeated doses of growth factors does increase the costs of stem cell-based therapy and might be associated with several side effects including synovial fibrosis, osteophyte induction, and other osteoarthritic-like symptoms [62, 63].

### 2.2. Induced Pluripotent Stem Cells

Part of the issues associated with MSCs can be circumvented by using iPSCs. iPSCs are an ideal patient-specific unlimited cell source for autologous tissue regeneration. Promising *in vitro* results have already been demonstrated in the cartilage engineering field for iPSCs generated from various cell types [20, 21, 23, 64, 65]. Nevertheless, Guzzo et al. stressed the influence of cell type origin on their chondrogenic capacity, where superior properties could be assigned to iPSCs from chondrogenic origin [66], which may be due to the preservation of the epigenetic memory [67].

Analogous to MSCs, indirect cocultures of iPSCs with primary chondrocytes could directly induce the formation of chondrocytes [20]. Furthermore, iPSCs could be committed to the chondrogenic lineage in high-density pellet culture systems, enhanced by the addition of growth factors from the TGF-*β* superfamily. Nevertheless, the resulting cartilage is a heterogeneous combination of hypertrophic, articular, and fibrocartilage [68]. This heterogeneity could be reduced by first differentiating iPSCs towards an intermediate cell population, such as MSCs [68, 69] or embryonic cell types [23, 65, 70]. An alternative approach to further enhance the chondrogenic potential is seeding iPSCs into scaffolds [71].

Although iPSCs express higher proliferation rates [72] and similar or superior chondrogenic differentiation potential [14, 64] compared to MSCs, other limitations remain associated with these stem cells. Patient-specific autologous iPSC generation and transplantation are very expensive. Allogeneic therapy would be more attractive, but immune rejection cannot be excluded [73]. Analogous to MSCs, it remains uncertain whether the regenerated cartilage induced by iPSCs preserves the mechanical and functional properties of native articular cartilage. Furthermore, also for iPSCs, the presence of hypertrophic signals under *in vitro* conditions, even though to a lesser extent than for MSCs, might indicate the formation of low-quality cartilage tissue by iPSCs [14, 23]. Safety issue is the most important concern that hampers their general use [74]. The potential reactivation of pluripotency in iPSCs or iPSC-derived chondrocytes should be addressed [75]. Moreover, when using retrovirally transduced iPSCs, where the retroviral gene is integrated in the host, a higher risk for teratoma formation in cell transplants is reported [76]. Therefore, adequate phenotyping of (fully) chondrogenic committed iPSCs is needed before transplantation of cells in (pre)clinical use. Several approaches have been proposed to develop iPSCs with a lower risk for tumorigenicity [69, 75, 77–79]. Nakagawa et al. generated iPSCs without Myc from mouse and human fibroblasts and reduced the tumorigenicity of cells [77]. Fusaki and colleagues induced transgene-free human pluripotent stem cells by means of a vector based on the Sendai virus, which does not integrate into the host [78]. Nejadnik et al. used the integration- and viral-free minicircle reprogramming technique to reduce the reactivation of pluripotency in the used human iPSC-derived chondrocytes [69]. Alternatively, transgene-free iPSCs can be used as generated by Wu and colleagues [80]. Additionally, iPSC-derived chondrocytes could be engineered to express a suicide gene in order to eliminate the cells, which was reported to be efficient in ESCs and BM-MSCs [81, 82].

## 3. Mechanisms of Action of Stem Cell-Based Therapies for Cartilage Regeneration

**Figure 1:**
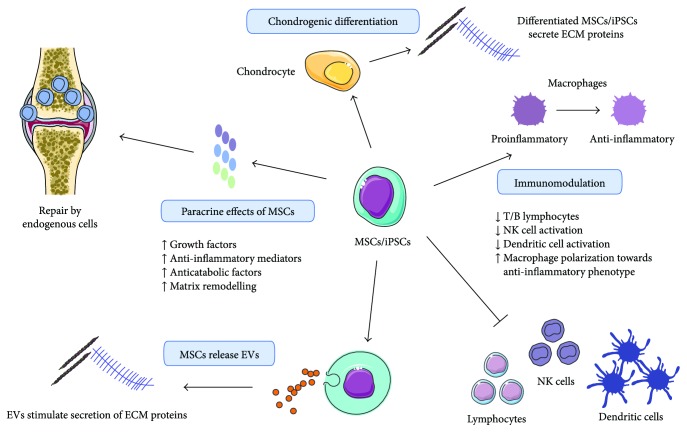
Mechanisms of action of stem cell-based therapies in cartilage regeneration. First, stem cells could be applied as cell replacement therapy because of their chondrogenic differentiation potential. Differentiated mesenchymal stem cells (MSCs) and induced pluripotent stem cells (iPSCs) secrete proteoglycans and collagen II. Secondly, it is suggested that the tissue is regenerated by endogenous cells under the influence of paracrine factors secreted by stem cells. Extracellular vesicles (EVs) contribute to stem cell-mediated cartilage regeneration by promoting the formation of new cartilage and the deposition of collagen II and GAGs. Finally, immunomodulatory effects are also observed. *This image was created using Servier Medical Art.*

Stem cell-based therapies were initially developed as a cell replacement therapy due to the chondrogenic differentiation potential of stem cells [14, 23, 52, 83, 84]. Moreover, differentiated MSCs, ESCs, and iPSCs secrete PGs and collagen II [23, 85–88] which are essential components of cartilage tissue. However, it has been shown that upon intra-articular transplantation, MSCs induce cartilage replacement, but the principal source of repair tissue is derived from endogenous cells [89]. Therefore, it is postulated that the paracrine effect of the transplanted cells on the damaged host environment is mainly responsible for stimulating cartilage regeneration (Figure 1). MSCs that were exposed to tumor necrosis factor alpha (TNF-*α*) and IL-1*β* were shown to upregulate the expression of several growth factors, anti-inflammatory mediators (*vide infra*), and anticatabolic factors ultimately leading to (stem) cell-mediated cartilage regeneration (reviewed in [90, 91]). The main growth factors associated with cartilage regeneration that are secreted by MSCs belong to the TGF-*β* superfamily [92]. Moreover, adipose tissue-derived mesenchymal stem cells (AT-MSCs) were demonstrated to diminish MMP-13 expression upon transplantation, potentially counteracting collagen degeneration in pathological cartilage [93]. In addition to the paracrine effect of soluble factors, extracellular vesicles (EVs), released by MSCs, have been shown to influence cartilage regeneration (Figure 1). Reports on stem cell EV-mediated cartilage repair are scarce. Studies showed that MSC-EVs promoted the formation of new cartilage and the deposition of collagen II and glycosaminoglycans (GAGs) [94]. Additionally, EVs from MSCs that overexpressed miR-140-5p stimulated chondrocyte migration and proliferation [95]. Moreover, it was recently reported that BM-MSCs secrete hyaluronan- (HA-) coated EVs [96], which may allow MSC homing to cartilage defects in a receptor-mediated way via CD44. Although stem cell EVs have shown beneficial effects in cartilage repair, it should be noted that EVs may also have damaging effects in arthritis [13].

Furthermore, it has been demonstrated that MSCs possess immunomodulatory properties (Figure 1) [97]. Given the immune component underlying cartilage degeneration, modulating the immune response might contribute to reducing cartilage loss in diseases where an uncontrolled immune response is detrimental [98, 99]. BM-MSCs, for example, have been shown to suppress T-cell proliferation [100, 101] and to induce T-cell apoptosis [102]. The resulting debris stimulated phagocytes to produce TGF-*β* which increased the number of regulatory T cells [102]. Moreover, T-cell proliferation was inhibited by BM-MSCs that produced prostaglandin E2 (PGE2) and indoleamine 2,3-dioxygenase (IDO) [103, 104]. These factors were also shown to inhibit NK cell activation [105]. Also, MSCs derived from the dental pulp possess immunomodulatory properties [106, 107]. The proliferation, activation, maturation, and antigen presentation of dendritic cells were also inhibited by MSC subtypes [108–112], and macrophage/microglia polarization was shifted towards an anti-inflammatory phenotype after exposure to MSCs, their secretome, or EVs [110–115]. Additionally, MSCs were able to modulate the B cell response by paracrine actions [116, 117]. Next to MSCs, iPSC- or ESC-derived MSCs could also inhibit lymphocyte proliferation and function [118–121] and NK cell function [120].

## 4. The Importance of a Translational Animal Model and Appropriate Outcome Measures

While *in vitro* studies and models offer a substantial amount of information about the potential of stem cells for cartilage repair [122, 123], more in-depth knowledge about their behavior *in vivo* should be derived from immunocompetent animal models [124]. In orthopedic research, to move new technologies from bench to bedside, strict preclinical studies using translational animal models are required [125]. Preclinical studies evaluating the healing of cartilage defects have been performed using both small and large animal models including murine, lapine, porcine, caprine, ovine, canine, and equine models [16, 124]. The following section will focus on the advantages and disadvantages of utilizing small and large animals for cartilage repair studies as well as some key factors in study design and the usage of validated outcome measures.

### 4.1. Choice of Animal Model: Small versus Large Animal Models

Articular cartilage defects have been created in small animals, such as mice [84], rats [126–129], and rabbits [130–132]. Smaller animal models are cost-effective and easy to house, and rodents are available in a variety of genetically modified strains with minimal biological variability [9, 124]. However, the small joint size, the thin cartilage [133, 134], altered biomechanics [135, 136], and increased spontaneous intrinsic healing [137] hamper the study of the regenerative capacity of stem cells and these mechanisms of healing cannot be fully extrapolated to human cartilage repair [9, 124]. Rodents have mainly been used to assess chondrogenesis of cell-based therapies by subcutaneous [138], intramuscular [139], and intra-articular [140] implantations of cells [9]. Of all small animals, the rabbit model is the most utilized model in cartilage regeneration studies because of the slightly larger knee joint size in comparison to rodents [16]. Despite their limited translational capacity, small animals can be very useful as a proof-of-principle study and to assess therapy safety before moving on to preclinical studies using larger animals [9, 125].

Large animal models play a more substantial role in translational research because of a larger joint size and thicker cartilage; however, their preclinical use is often hindered by high costs and difficulties in animal handling. A variety of large animal models have been used to investigate cartilage repair strategies, including horses [141–143], dogs [144], sheep [145–149], goats [150, 151] and (mini)pigs [152–155], each with their own strengths and limitations.

The knee anatomy [156–158], cartilage thickness [133, 159], biomechanical loading environment [124] and the subchondral bone properties [136] of the above-mentioned species differ variously from the human condition [124, 160]. An advantage of using the porcine model is the cartilage thickness of 1.5 mm–2 mm, compared to human cartilage thickness of 2.4 mm–2.6 mm [152, 159]. Dogs, in contrast, have thinner cartilage (0.95 mm–1.3 mm) compared to human cartilage [124, 159]. For the goat, cartilage thickness has been reported between 0.8 mm and 2 mm, whereas cartilage thickness in sheep ranges from 0.4 mm to 1.7 mm [124, 159]. Of all animal models used in cartilage regeneration studies, the horse's cartilage thickness (1.75 mm–2 mm) provides the closest approximation to the human situation [133, 136, 159, 161].

In a comparative anatomical analysis, the goat stifle displayed strong anatomic similarities to the human knee except for a long trochlear groove with medial and lateral ridges and the intercondylar notch width [124, 156]. According to Osterhoff et al., the ovine stifle is very similar to the human knee except for the femoral intercondylar notch width, the patellofemoral joint's biomechanics, and the proximal tibia's cortical bone stock [158]. More recently, Vandeweerd and colleagues described several anatomical features in the ovine stifle [157]. Although the goat and ovine stifles are very similar to the human knee, these few anatomical differences remain and should be taken under consideration when selecting them as a suitable animal model [156–158], which, for instance, can have an impact on the volume of the synovial cavity. In addition to similar knee anatomy, the caprine model has been reported to have similar stifle biomechanics compared to human knees [124, 162]. While the horse model offers defect sizes comparable to human defect dimensions, the increased weight and the fact that the horse spends much of its time in standing position place defects under significant loading and this continuous loading cannot be diminished [159]. Nevertheless, this constant loading environment in the horse stifle joint could be argued to be beneficial for translational cartilage repair studies since the human knee provides a less challenging load environment [163].

Moreover, since numerous repair strategies rely on the subchondral repair mechanisms, subchondral bone properties must be considered when selecting the appropriate repair model [136]. According to Chevrier et al., the subchondral properties of the rabbit trochlea are similar to the human medial femoral condyle (MFC) [136]. The goat offers advantages in subchondral bone consistency, thickness, and trabecular structure, which are more similar to the human structure in comparison to either small animals, ovine models, or canine models [9, 124]. A major disadvantage of the ovine and equine models is the dense and hard subchondral bone, while the caprine model has a softer subchondral bone [9, 159]. In addition, subchondral bone cysts in sheep [145, 164] and goat [165] have been reported when the subchondral bone is involved in cartilage repair mechanisms [166].

Ultimately, when selecting the best repair model, comparable anatomy and joint function are not the only important aspects, but other factors need to be taken into consideration when performing translational preclinical studies (Table 1). A factor requiring major consideration is the choice of defect location [124]. Clinically, most defects are made on the femoral condyles or the trochlear groove [160]. However, defect position influences cartilage repair response as demonstrated in caprine and ovine models leading to contradictory results [147, 162]. These differences in repair potential are due to differences in cartilage thickness, loading mechanics, and subchondral bone properties within the knee and between species [136, 147, 162]. In addition, defects may occur where higher loads are expected [167]. Ideally, these areas should be used when defects are induced. Therefore, it is important to identify the prevalence of naturally occurring defects in animal models and to assess where the lesion should be created based on the biomechanics of the joint of the animal [124, 167]. The ovine model is a well-documented model, where the most frequent naturally occurring cartilage defects in the ovine knee occur on the axial aspect of medial tibial condyle (MTC) and on the MFC [167]. Critical size chondral and osteochondral defects have been reported in rats, rabbits, dogs, (mini)pigs, sheeps, goats, and horses (as shown in [125, 159, 168]). Skeletal maturity and animal age also affect repair mechanisms of cartilage defects, especially when the subchondral bone is fractured for induction of repair [136, 137, 166, 169, 170]. Experimental models in animals that have reached skeletal and articular cartilage maturity are needed before the effect of any novel regenerative strategies on adult cartilage repair can be clinically evaluated. According to the International Cartilage Repair Society (ICRS) recommendations, selection of the age of an experimental animal should be based on cartilage maturity rather than on skeletal maturity (closure of the growth plate) [166]. Cartilage maturity can be defined as the time point where a cartilage defect is not spontaneously repaired and at the presence of a well-defined zonal architecture, an intact continuous layer of calcified cartilage, and minimal vascular penetration in the subchondral bone plate [166]. This would confirm that the articular cartilage has the adequate cellular, biomechanical, and biochemical properties. Therefore, in preclinical cartilage repair studies, animals at the age of cartilage maturity, defined based on the aforementioned conditions, should be used (Table 1) [166].

While the choice of animal age, critical defect dimensions, and location in preclinical studies is often justified, gender selection is frequently overlooked. Regenerative strategies to address cartilage lesions and osteoarthritis have not sufficiently considered possible gender differences [171]. Therefore, potential gender effects must be taken more into consideration during analysis. Epidemiological studies demonstrated the presence of sex differences in osteoarthritis prevalence and incidence with females being at a higher risk to develop more severe knee osteoarthritis after reaching menopausal age [171]. Several researchers examined the role of sex hormones in osteoarthritis, including in ovine and murine models [167, 172–174]. Ma and colleagues showed that sex hormones, both testosterone and oestrogen, have a crucial influence on the advancement of osteoarthritis in mice. Testosterone aggravated the disease in male mice evidenced by the fact that orchiectomized mice showed a less severe osteoarthritis than intact males. Healthy female mice showed less severe osteoarthritis than ovariectomized females, demonstrating the protective role of female hormones [174]. In a biomechanical study in sheep, ovariectomy in females induced a detrimental effect on the intrinsic properties of the articular cartilage in the knee [172]. In human subjects, differences in knee joint volume and articular surface areas between men and women have been described [175]. Moreover, gender differences in cartilage composition and gait mechanics in young healthy, middle-aged healthy, and osteoarthritis cohorts are reported [176]. These differences might influence functional outcome after repair [177]. Thus, effective and well-designed regenerative preclinical studies are required and should lead to a better understanding of gender-specific differences in the mechanisms involved in cartilage re- and degeneration. Since osteoarthritis and cartilage biology are reported to be sex-dependent, the inclusion of female animals is essential for preclinical cartilage repair studies. If both sexes are included, an equal number of males and females per study group with short ranges of ages should be used. Moreover, results should be reported for both genders and per study group [171]. In addition, for large animals, it is more difficult to manage male animals, since sexual behaviour and mounting may increase loads on high limbs.

Obviously, the recommended study duration for evaluating cartilage repair in preclinical animal models is different for proof-of-concept or pilot studies (<6 months) versus late-stage preclinical studies in large animal models (>6 months) [124, 125, 166]. However, for late-stage preclinical studies, caution must be exercised when the study ends within a year or when no interval follow-up investigations are implemented since the repaired tissue can vary at earlier phases of healing and the sustainability of the repaired tissue is time-dependent [148, 153, 166]. Follow-up methods of noninvasive imaging are necessary [178, 179]. Ovine models allow for imaging techniques such as magnetic resonance imaging (MRI) [166, 180], while the equine model is much more difficult, or impossible, due to the size of animal versus size and costs of high-field MRI. Furthermore, the nature of the regenerative strategy, such as the use of autologous or allogeneic cell therapy, also needs to be considered. Other key issues in cartilage repair models are the choice of bilateral versus unilateral surgery and acute versus chronic defects [148, 166]. Bilateral repair models are suitable to minimize interanimal variability and to increase the number of treated limbs but are only useful if the treatments are not reciprocally influencing the opposite limbs [181]. Unilateral models, in contrast, ensure that the treatment is not influenced by the contralateral technique. In addition, these models allow easier joint immobilization and are exposed to less initial weight bearing on the operated limb. More importantly, unilateral models permit better evaluation of locomotion, range of motion, and gait [166].

The choice of animal model is also influenced by practical aspects such as ethical considerations, costs, and availability of housing accommodations, materials, and competent personnel [160]. Nowadays, it is increasingly difficult to obtain ethical permission for the usage of dogs and horses, while working with reformed sheep or goats is much easier to justify. Surgical limitations, such as the ability of the animal to tolerate anaesthesia and postsurgical recovery protocols or the possibility of second-look access, could influence the choice of a specific animal model [141, 142, 166, 182]. The ovine model, for instance, is particularly easy to handle, cost-effective, and easy to anaesthetize.

### 4.2. Follow-Up and Outcome Measures

Preclinical animal studies analyzing the capacity of new technologies in cartilage regeneration frequently suffer from a lack of noninvasive follow-up and outcome measures and are therefore often forced to use endpoint outcome measures such as histology and destructive mechanical testing (Table 1). Additionally, there is an increasing need for standardized technologies with a diagnostic significance over the whole defect and adjacent tissues, while incorporating reflections of costs, care, and ethics and mimicking the clinical investigations in human clinical trials [166, 178].

For longitudinal *in vivo* studies, it is advised to assess the animal at baseline and at different time points. Depending on the animal, healthy joint status at the start of the study should be evaluated via diagnostic imaging modalities since variability in cartilage thickness, bone structure, and the prevalence of naturally occurring cartilage defects and other lesions associated with osteoarthritis can occur among species [167, 183–185]. More specifically, spontaneously occurring cartilage lesions have been described in canine, equine, and aging ovine models [9, 166, 167]. Canine and equine models should be screened for naturally occurring osteoarthritis, since they can have lesions associated with osteoarthritis or osteochondritis dissecans [9, 166]. Noninvasive imaging of articular cartilage defects can be performed by magnetic resonance imaging (MRI) [186–188] or computed tomography arthrography (CTA) [185, 189, 190]. CTA has been shown to be more accurate than MRI to detect cartilage defects in humans [185, 191]. Recently, Hontoir et al. described CTA to be an accurate imaging method for detecting articular cartilage defects in the ovine stifle [185]. Additionally, the same authors compared the sensitivity and specificity of 3-Tesla (3-T) MRI and CTA to identify structural cartilage defects in the equine metacarpo/metatarsophalangeal. Hontoir and colleagues showed that CTA is superior to MRI due to its shorter acquisition time, enhanced correlation to macroscopic assessment, and its specificity and sensitivity in identifying articular cartilage defects; nonetheless, MRI has the advantage to assess soft tissues and subchondral bone [189].

For the visualization of cartilage, diagnostic imaging techniques such as ultrasound, computed tomography (CT), and MRI can be used [125, 178]. More recently, novel quantitative MRI and CT techniques are being adopted as outcome measures after cartilage repair [178, 188, 190]. Compositional imaging MRI is being progressively applied to assess the biochemical composition of cartilage for the longitudinal follow-up of cartilage repair studies [179]. More specifically, T2 mapping combined with delayed gadolinium-enhanced MRI of cartilage (dGEMRIC) seems to be a good compositional imaging modality to monitor cartilage repair and to discriminate between a collagen network with zonal organization and healthy cartilage [179, 192]. Combining multiple imaging techniques may yield a better understanding of both the collagen and PG content of the repaired defect [193]. T2 mapping provides information about the interaction of water molecules and the collagen network, while dGEMRIC evaluates GAG concentration within the cartilage [194]. In human patients, Kurkijarvi et al. demonstrated that combining datasets from dGEMRIC and T2 relaxation time mapping provides additional information on cartilage repair [192]. Recently, T2 mapping and dGEMRIC were used for assessing cartilage repair after allograft chondrocyte implantation in a rabbit model, where dGEMRIC data showed a high correlation with histological and biochemical data [194]. In goat models, T2 mapping and dGEMRIC have also been used as outcome measures in a study evaluating cartilage repair after microfracture in an osteochondral defect of both the medial and lateral femoral condyles [195]. Alternatively, T1ρ has been used as a complementary imaging tool to T2 mapping which allows for the examination of PGs and the collagen organization [179]. However, one of the major issues of using T1ρ is reaching an adequate resolution with an acceptable acquisition time [179]. More recently, van Tiel and colleagues showed that dGEMRIC is more robust in accurately measuring cartilage GAGs *in vivo* in patients compared to T1ρ mapping [196].

Although substantial progress has been made in real-time *in vivo* cartilage imaging, spatiotemporal tracking of stem cells *in vivo* using MRI, bioluminescence imaging (BLI), fluorescence imaging (FLI), or nuclear imaging methods should be the focus when developing novel imaging techniques [178]. Superparamagnetic iron oxide (SPIO) particles are used for cartilage tissue engineering to monitor transplanted cells [197, 198]. However, SPIO particles are associated with several drawbacks such as the inability to distinguish viable cells from dead cells and from cells engulfed by phagocytes [199]. One of the possibilities to minimize particle transfer to other cells is the use of reporter genes. BLI compatible reporter genes such as red/green luciferases have already been used for cartilage tissue engineering to track transplanted cells [200]. In addition, by labeling cells with an additional chondrogenic reporter gene, cell differentiation can be monitored by means of dual bioluminescence labeling [201]. While this optical imaging method offers a sensitive technique to track stem cells, its use in larger animal models is limited because of a loss of signal intensity from deeper tissues due to scattering [202].

At baseline and at longitudinal intervals, clinically relevant examinations of cartilage repair and functional improvement should be carried out. These should be performed by a veterinary surgeon familiar with observing clinical signs and locomotion by assessment of changes in joint palpation, quantitative monitoring of pain, and changes in joint function or locomotion by gait analysis [125, 166, 203–206]. In rats, several scoring systems have been published to measure lameness, stride length and limb rotation, dynamic force application, and hind limb motion [206]. Moreover, for large animal models, kinematic marker analysis, ground reaction force measurements, and observational gait assessment have been progressively used in osteoarthritis-related gait alterations in canine, ovine, and equine models [206]. Several scaling systems have been documented in the literature, such as the American Association of Equine Practitioners (AAEP) lameness scale in the horse ranging from zero to five [207]. In ovine models, a numeric ranking scale can be used to determine comfort, movement, and flock behaviour [204]. A more detailed lameness scoring system has been published by Kaler et al. ranging from “normal” (0) to “unable to stand or move” (6) [203]. Overall, clinical assessment and gait monitoring are indispensable in order to increase the translational value of preclinical animal studies to human clinical trials and to the clinic.

Biomarkers represent an additional tool to evaluate normal and pathological processes or to evaluate the interventional repair strategies [208, 209]. These biomarkers may be identified and quantified via enzyme-linked immunosorbent assays (ELISA) or other protein assays in synovial fluid or other biological fluids such as in the blood and urine [208, 209]. Synovial and other biological fluid collections should be performed at baseline and multiple time points [166], since synovial fluid biomarkers have the capacity to reflect the articular environment before treatment and could possibly inform on postoperative outcomes [208]. In small animal models, however, it can be difficult to obtain sufficient amounts of biological fluid at multiple time points necessary for biomarker analysis [210]. To solve this, the use of paper or alginate to obtain small amounts of synovial fluid has been described to be successful and effective [211]. Because of the relatively larger joint size in large animal models, a collection of synovial fluid and serum biomarkers can be more easily performed [161]. Nevertheless, a major difficulty to perform repeated collections is the increased inflammation in the joint due to iatrogenic damage. Biomarkers of particular interest are markers for cartilage or synovium metabolism or markers involved in pathological pathways, such as inflammation [209]. Recently, biological (synovial) fluid markers in osteoarthritis were thoroughly reviewed by Nguyen and colleagues [209]. Besides analyte quantifications to assess changes in inflammation and cartilage turnover, volume and physical characteristics of the synovial fluid, such as viscosity, could also be used as an outcome measure in preclinical studies [166].

At the end of *in vivo* studies, cadaver tissue can undergo ex vivo high-resolution MRI [212, 213] and *μ*CT [214] to evaluate structural improvements. Hereafter, macroscopic/arthroscopic scoring, histological and histomorphometric scoring methods, quantification of collagen and GAG expression by immunohistochemistry, collagen organization by polarized light microscopy and subchondral bone, and adjacent tissue integration are all outcome methods that should ideally be performed [214–218].

Nowadays, many histological scoring systems are available, contributing to the confusion on the use of an appropriate scoring method for a specific research question and study settings [219]. Moreover, it is unclear which scoring systems are validated and how study results can be compared between studies using different scoring methods [219]. The variety of histological scoring systems for the analysis of normal or osteoarthritic *in vivo* repaired or *in vitro* tissue-engineered cartilage was thoroughly reviewed by Rutgers et al. [219]. Normal cartilage can be distinguished from osteoarthritic cartilage via the Histological-Histochemical Grading System (HHGS) or HHGS-related systems and the Osteoarthritis Research Society International (OARSI) scoring method [219]. Of the various scoring systems available for the analysis of *in vivo* repaired cartilage, the ICRS II score seems most suitable in humans. In preclinical cartilage repair studies, the validated Pineda score or O'Driscoll score is advisable [219]. Other histological scoring systems for preclinical cartilage repair are widely used. In addition to the Pineda score, the Wakitani score is an elementary scoring system, reflecting not more than five parameters [220]. The Pineda score assesses four histological parameters: cell morphology, matrix staining, lesion filling, and osteochondral junction [220]. The O'Driscoll score is a more complex histological scoring method which also assesses surface regularity, structural integrity, cellularity, chondrocyte clustering, adjacent bonding, and adjacent cartilage degeneration. In addition to the O'Driscoll score, also the Fortier and Sellers scores are more comprehensive scoring systems [220]. Orth et al. showed that both elementary and comprehensive histological scoring systems are appropriate to quantify articular cartilage repair [220]. However, complex scoring systems provide more descriptive data about the character of the repair tissue [220]. The use of validated scores, such as the Pineda score or the O'Driscoll score, may significantly increase comparability of information and should thus stimulate consistency between studies. Importantly, histological and biochemical evaluations are complementary tools to assess experimental articular cartilage repair *in vivo* [219]. A key goal of regenerating mature cartilage tissue is to regenerate a tissue with biochemical/biomolecular and mechanical properties resembling those of native cartilage tissue. Small biopsies for biochemistry (water content, GAGs/PG content, and collagen content) and/or biomechanical testing should ideally be gathered before fixation of the repaired tissue for histology [217]. In addition to typical end-point destructive measures to assess mechanical properties, indentation testing provides a nondestructive compressive technique for in situ mechanical evaluation [178, 221]. Large animal models allow the harvest of a large amount of repaired tissue in order to have parallel histological, biochemical, and biomechanical analyses of the repaired area postmortem [166, 222].

Finally, the combined utilization of *in vivo* clinical tests and assessment of locomotion, *in vivo* noninvasive imaging methods, and postmortem evaluation of tissue structure with validated scoring systems, biochemical composition, and mechanical properties will deliver a robust outcome analysis in order to improve the translational value of animal models in cartilage repair.

## 5. *In Vivo* Evidence of Stem Cells in Cartilage Regeneration

Within the field of cartilage regeneration, numerous preclinical studies have been published demonstrating the favorable effects of cell-based approaches on the repair of cartilage defects. Although the cartilage contains an inherent progenitor cell population [223–225], to our knowledge, robust scientific reports describing their *in vivo* regenerative potential in particular defects are currently lacking. Given their aforementioned *in vitro* properties, certain pluripotent and multipotent stem cell populations are considered to be credible candidates for stem cell-based repair and regeneration of cartilage tissue. iPSCs, for example, have been shown to successfully repair cartilage defects in a variety of rat models, following predifferentiation towards a chondrogenic lineage [14, 69, 226]. However, due to their pluripotent nature, the use of these stem cells still bears the risk of tumorigenesis [1]. Saito and coworkers, for instance, reported the formation of an immature teratoma in one animal, following a prolonged transplantation period of predifferentiated iPSCs in the knee joints of immunocompromised mice [74].

With regard to multipotent stem cell populations, one of the most frequently applied stem cell sources in the repair and regeneration of articular cartilage defects are MSCs. BM-MSCs in particular have been used in a wide variety of small and large animal models [16, 227]. Zhang et al., for example, recently demonstrated the regeneration of meniscal tissue after transplantation of BM-MSC-seeded poly(*ε*-caprolactone) (PCL) scaffolds in rabbits [228]. Formation of hyaline-like cartilage tissue was also observed after the treatment of a canine osteochondral defect with autologous BM-MSCs [144]. Sridharan and coworkers reported the successful repair of a rat trochlear knee defect after transplantation of high density BM-MSC/fibrin aggregates [229]. Similar results were found by Itokazu et al., indicating osteochondral repair in nude rats after transplantation of human BM-MSC cell sheets [230].

Although BM-MSCs are reported to have a predominantly positive effect on cartilage repair and regeneration, their invasive collection as well as the limited yield of stem cells during this procedure encourages the search for alternative tissue sources of MSCs [231, 232]. Substantial amounts of AT-MSCs, for example, can be relatively easy to be obtained through liposuction, and their intrinsic behavior does not seem to be affected by donor-related characteristics, such as age [231, 233]. Recent work of Mehrabani and coworkers demonstrated the successful formation of hyaline cartilage tissue after intra-articular injection of AT-MSCs in the knee joints of rabbits [234]. Implantation of scaffold-free spheroids of AT-MSCs into an osteochondral defect in two adult (mini)pigs led to the regeneration of the original cartilage tissue [235]. While hypoxic preconditioning of AT-MSCs had no effect on their *in vivo* chondrogenic potential [236], pretreatment of these stem cells with activated platelet-rich plasma substantially improved articular healing after transplantation in immunocompromised mice [237]. However, in comparison to BM-MSCs, AT-MSCs exhibit a significantly lower osteogenic and chondrogenic differentiation potential both *in vitro* and *in vivo* [55, 238–240].

Synovium-derived MSCs, on the other hand, not only display a higher proliferation potential in comparison to other sources of MSCs but also show a more pronounced production of cartilage-specific ECM when transplanted into an osteochondral defect in rabbits [238, 241, 242]. Similar findings were reported by Nakamura and coworkers, indicating the successful formation of cartilage tissue after intra-articular injection of allogeneic synovium-derived MSCs in the knee joints of pigs [243].

With regard to the mechanisms underlying the favorable effects of MSCs in cartilage repair, it remains unclear whether chondrogenic differentiation is a necessary prerequisite for cartilage tissue engineering as an increasing amount of evidence suggests that both the secretion of paracrine factors and the subsequent attraction of resident cells can also mediate tissue regeneration [7, 244]. In order to promote these complex interactions, MSCs may be combined with chondrocytes in coculture systems, supported by exogenous growth factors and/or biomaterials to recreate the most optimal microenvironment for cartilage repair and regeneration [245]. Sabatino et al., for example, reported the successful production of cartilage grafts in a proof-of-principle mouse model. After subcutaneous transplantation of (precultured) collagen sponges containing BM-MSCs and articular chondrocytes, an increased GAG and collagen type II content was observed [246]. Similar results were found by Cai et al., indicating the formation of cartilage-specific ECM after subcutaneous transplantation of AT-MSCs and auricular chondrocytes supported by Pluronic F-127 [247]. In addition to subcutaneous transplantation, cocultures have also been directly applied in cartilage defects. Successful regeneration of meniscus tissue was demonstrated after transplantation of a polyvinyl alcohol/chitosan scaffold containing an AT-MSC/chondrocyte coculture in New Zealand rabbits suffering from a unilateral, medial meniscectomy. However, no significant differences were observed between the coculture scaffolds and the scaffolds merely containing articular chondrocytes [248].

In terms of delivery methods, numerous researchers used a scaffold-free intra-articular injection of stem cells. Nam and colleagues conducted a pilot study to test the effects of an intra-articular injection of autologous mesenchymal stromal cells on the repair outcomes of bone marrow stimulation (BMS) surgery in a caprine model. Results showed that the intra-articular injection of BM-MSCs following BMS intervention induced better cartilage repair outcomes [150]. In another study, MSCs were injected with hyaluronic acid (HA) and this resulted in good defect coverage at 12 weeks postinjection in a pig model [249]. The major advantage of an intra-articular injection of stem cells is the simplicity of the administration, but it would only be useful in early stages of cartilage injury. Additionally, intra-articular injection can lead to cell dispersion and an insufficient amount of cells reaching the defect required for repair [227]. One way to solve this is by using a local adherent technique for transplanting MSCs to the cartilage defect. Koga et al. showed in a pig model that placing an MSC suspension on the cartilage lesion for 10 minutes resulted in adherence of more than 60% of cells to the defect and induced cartilage regeneration [250]. Similarly, Nakamura and colleagues recently showed the same adherent technique with synovial MSCs in a pig model [243]. Unfortunately, by using these techniques, the transplanted cells lack an ECM, which makes it challenging to exploit the function of cells since the 3D environment is reported to be crucial in the processes of cell proliferation and differentiation [251]. To address this, a novel scaffold-free 3D tissue-engineered construct (TEC) has recently been developed, composed of native ECM, synthesized by MSCs [251]. In addition, MSCs seeded in acellular cartilage matrices/sheets also showed successful cartilage repair [252, 253].

Scaffolds are preferably biocompatible or biodegradable and can be implemented via a minimally invasive surgical procedure. Furthermore, they should provide rigid mechanical properties and offer some additional advantages such as adequate nutrient transport and adhesion to the defect [254]. Stem cells have been combined with a wide variety of natural and synthetic biomaterials to support and promote cartilage repair and regeneration [16, 231, 255–257]. This combinatorial approach has led to several successful *in vivo* applications of cell-seeded biomaterials for cartilage repair [14, 51, 52]. Of the various scaffold materials, the most commonly explored are hydrogels, which are cross-linked water-swollen systems [254]. Hydrogels gained a lot of interest because of their ability to homogeneously contain cells in a 3D environment and the minimal invasive injection procedure [254, 258, 259]. Natural hydrogels based on polysaccharides, such as chitosan, HA, alginate, and agarose, have been reported to support cartilage regeneration [254]. Recently, it was shown that MSCs isolated from the dental pulp cultured in an alginate scaffold successfully regenerated articular cartilage [260]. HA-based hydrogels are one of the most extensively used hydrogels in cartilage repair [254] and have been reported to improve cartilage specific-matrix deposition of MSCs [254, 259, 261]. In a direct comparative study in rats between several hydrogels such as alginate, pluronic, HA, and chitosan with human umbilical cord blood derived mesenchymal stem cells (hUCB-MSCs), the combination of hUCB-MSCs-HA resulted in superior cartilage repair on a macroscopic and histological level [262]. Similarly, combining hUCB-MSCs with a HA hydrogel promoted cartilage regeneration in an osteochondral defect minipig model [155]. With regard to natural biomaterials and hydrogels based on proteins such as collagen, gelatin, fibroin, and fibrin, Wilke et al. described an early chondrogenic response after intra-articular injection of a fibrin gel containing BM-MSCs in horses [142]. Fibrin is a commonly used natural protein with chondrogenic-inducing properties [254, 259]. However, one of the major disadvantages of using fibrin gels is the fast degradation [263], resulting in less beneficial results *in vivo* [264]. More recently, platelet-rich fibrin (PRF) has gained more interest to provide a 3D environment for stem cells, consisting a strong fibrin network and supportive platelets. PRF has the ability to support the proliferation of MSCs and favors cytokine enmeshment and cellular migration [144]. Kazemi and colleagues showed that the use of BM-MSCs seeded on PRF could be a novel method for articular cartilage regeneration, where the PRF creates a suitable environment for stem cell proliferation and differentiation by secreting growth factors [144]. Alternatively, collagen is abundant in native articular cartilage and is therefore widely used in preclinical animal studies as a stem cell carrier [254]. The application of collagen combined with BM-MSCs led to fully repaired cartilage tissue in a porcine model [265]. However, the softness of collagen gels is one of the major concerns for *in vivo* cartilage repair [254]. Recently, a natural type II collagen hydrogel, fibrin sealant, and adipose-derived stem cells have been recommended as a positive combination for articular cartilage repair in rabbits [266]. In addition, transplantation of synovium-derived MSCs in a combination of collagen type I/HA/fibrinogen composite gel induced the formation of hyaline cartilage tissue in a lapine osteochondral defect model [242]. One of the major limitations of natural hydrogels is the low mechanical strength [259], which needs further modifications or combinations with other natural or synthetic polymers (composite scaffold). A number of advantages were also reported for synthetic polymers, such as a controlled degradation and good mechano-physical properties [267]. Poly-(lactic-(co-glycolic)) acid (PL(G)A), for example, is one of the most widely applied synthetic scaffold materials in stem cell-based cartilage tissue engineering [16]. Recent work of Yin et al. showed the regeneration of articular cartilage after transplantation of a TGF-*β*1-immobilized PLGA scaffold seeded with AT-MSCs into a full-thickness cartilage defect in New Zealand white rabbits [268]. Similar findings of successful cartilage engineering were reported earlier for BM-MSCs, indicating the successful formation of hyaline cartilage tissue by connective tissue growth factor- (CTGF-) modified stem cells contained within a sodium hydroxide-treated PLGA scaffold [269]. Caminal et al., however, only demonstrated a transient improvement caused by BM-MSC-seeded PLGA scaffolds in sheep with a critical size chondral defect [270]. Recently, hiPSCs-MSCs were seeded onto a PLGA scaffold and transplanted into a cartilage defect in a rabbit model. Results showed that the hiPSCs-MSCs-PLGA scaffold experimental group had the potential to repair cartilage defects *in vivo* [132]. One of the major disadvantages of using such synthetic polymers is the possibility to elicit an immune response [267]. Moreover, synthetic biomaterials lack biocompatibility and biological activity [259]. As previously mentioned, it is better to combine a synthetic polymer with a natural polymer to improve biological activity [259]. Many other scaffold materials have been tested in an attempt to improve cartilage repair by using MSCs. A wide overview of most used scaffolds can be found in the review article published by Goldberg and colleagues [16].

In order to make human stem cells applicable to human clinical translation, more in-depth knowledge about their *in vivo* behaviour should be derived from larger immunocompetent animals. Several researchers used human MSC transplantation in smaller nonimmunosuppressed animals and reported no graft rejection [271, 272]. Transplantation studies in larger animal models such as immunocompetent dog and swine reported similar results and even point out immunosuppressive capacities which are related to the MSC transplantation [273, 274]. Dayan and colleagues demonstrated that transplantation of human MSCs in ovine immunocompetent animal models showed clinical safety and efficacy suggesting that immunocompetent sheep can serve as a suitable preclinical large animal model for testing human stem cells [275]. In the unfortunate case of detecting an immunogenic response following human stem cell transplantation into the animal model, generally known immunosuppressive drugs can be administered at the time of transplantation. Alternatively, autologous transplantation of stem cells can be considered. However, one of the disadvantages of using autologous stem cells isolated from larger animals is the lack of well-characterized species-specific stem cells and protocols for their culturing and differentiation [276]. For autologous iPSCs in preclinical research in animal models, it appears that iPSCs in farm animals have not yet received the deserved attention [277]. Ogorevc et al. described only 32 studies addressing the development of iPSCs in pig, cattle, horse, sheep, goat, and rabbit [277]. In addition, large animal commercial products, such as antibodies, reagents, and microarrays, are not widely available [276]. Nevertheless, beyond any doubt, for translational research in cell therapy, testing human stem cells in preclinical animal models which are immunocompetent should gain more attention.

## 6. Conclusion

Despite the multiple promising mechanisms of action of stem cell-based therapies for cartilage repair, supported by advances in bioengineering and biomaterials to exploit the full potential of stem cells, it is not yet possible to achieve engineered cartilage possessing identical properties as native cartilage [17, 278]. Several concerns have to be addressed when considering these therapies for large-scale human translation.

Before moving to the clinic with a universally applicable therapy, issues involving the heterogeneity of MSC sources as well as the heterogeneity within MSC populations, isolation methods, and differentiation protocols should be addressed [24]. Other factors such as aging [279], serial passaging [61], and the presence of comorbidities in the donor [60] can restrict the chondrogenic differentiation potential of MSCs. Furthermore, MSCs mainly produce collagen type I, while the main collagen subtype in the cartilage is collagen II. This needs to be taken into account to avoid the production of fibrocartilage or ossified hypertrophic cartilage [280]. Biomaterials can be used in combination with stem cells in order to support and promote cartilage repair and regeneration [16, 278]. In the future, biomaterials can offer enhanced control of cell fate, enable sustained and localized release of paracrine factors, and facilitate remodeling of newly formed tissue [7].

Despite conflicting preclinical results, the use of allogeneic MSCs is gaining support as it avoids donor site morbidity and allows for single-stage procedures, thereby reducing the financial burden and increasing the simplicity of cell-based therapies [16]. Since different stem cell sources show inherent differences in their differentiation potential, secretome, and ECM profile, various MSC sources should be compared to select the most promising one for allogeneic therapies [7]. However, applying therapies involving allogeneic MSCs on a large scale requires cell banking possibilities and long-term safety and efficacy studies in order to assess possible immune rejection.

iPSCs have been explored as a possible alternative to MSCs due to their superior self-renewal capacity [1], proliferation rate [72], and chondrogenic differentiation potential [14, 64]. However, the most important obstacle in the use of iPSCs is the risk for teratoma formation after transplantation [1]. Therefore, before this technology can progress to clinical translation, research into the control of cell phenotype and cell fate is required in order to alleviate the concerns for tumorigenesis [16].

Under ideal circumstances, novel therapies would reach the market after *in vitro* data were used to inform preclinical studies, which in turn led to human clinical trials [16]. Researchers should be aware that every animal model is associated with its advantages and disadvantages and the choice of the model should match the research hypothesis and is important to ensure the translation to the clinic [9, 124]. Furthermore, the current lack of standardized protocols (i.e., cell delivery route and number of transplanted cells) as well as the wide variety of different outcome measures used to evaluate preclinical studies makes it difficult to draw definite conclusions regarding the potential use of stem cell-based approaches in cartilage tissue engineering through direct comparison of studies. Furthermore, gender differences in most animal studies have not been adequately investigated and should gain more attention. Moreover, the same applies for *in vitro* studies, where researchers, using primary cells or cell lines, often do not compare results between sexes. For cell lines, the gender of the cell line provided is frequently not mentioned, leading to conclusions which cannot be drawn for the whole population [171].

Despite these hurdles, at least 19 clinical trials have been registered using stem cell-based therapies for cartilage repair procedures [278]. Unfortunately, the quality of the existing clinical data is rather limited, but more recently registered clinical trials are showing improvement in the study design and methodology. This might in part be explained by the methodical recommendations developed by the ICRS [281]. This consensus statement includes guidelines for the statistical study design, patient recruitment, and considerations for appropriate control groups in order to help clinicians achieve high-quality data [281].

## Figures and Tables

**Table 1:** Key factors for the selection of a translational animal model for cartilage repair.

Aspect	Remark/recommendation
Anatomy and biomechanics	(i) Large difference in anatomy and biomechanics remains between animal models and humans

Cartilage thickness	(i) Large animals provide closer proximity to the human condition (ii) Depends on topographic location in joint

Subchondral bone properties	(i) Effect on repair mechanisms (ii) Depends on topographic location in joint

Defect dimensions and location	(i) Critical size chondral or osteochondral (ii) Location of defect influences cartilage repair (iii) Femoral condyles or trochlea (iv) Defect should be made based on the biomechanics of the joint of the animal

Age and gender	(i) Age and gender may have effect on repair mechanism (ii) Inclusion of skeletally mature animals with mature cartilage (human—near puberty) (a) Rabbit—8 months (b) Dog—24 months (c) Pig—18 months (d) Sheep—24 months (e) Goat—24 months (f) Horse—24 months (iii) Gender effects must be taken into consideration (iv) Use animals with short range of ages and with similar sex

Study duration	(i) Depends on type of study (ii) Proof-of-principle (<6 months) versus late-stage study (6 months–12 months)

Surgical and practical considerations	(i) Unilateral versus bilateral repair models (a) Unilateral models: evaluation of locomotion, range of motion and gait, better immobilization, and no influence of contralateral technique (b) Bilateral models: minimize interanimal variability (ii) Postoperative management should be tolerated (iii) Ethical permission for small animals and ruminants is easier to obtain (iv) Surgical feasibility must be taken into account (v) Financial costs to house and handle differ variously between animals (vi) Availability of facilities, competent personnel, and equipment

Validated outcome measures	(i) At baseline, *in vivo* and post mortem (ii) Clinical response and kinematics (iii) Biological fluid collection (iv) Noninvasive compositional imaging MRI (v) Ex vivo high resolution magnetic resonance imaging (MRI) or microcomputed tomography (*μ*CT) (vi) Tracking and monitoring (vii) Macroscopic/arthroscopic scoring (viii) Histological and histomorphometric scoring (ix) Mechanical testing (x) Biomolecular and biochemical testing
